# Unravelling the first key steps in equine herpesvirus type 5 (EHV5) pathogenesis using ex vivo and in vitro equine models

**DOI:** 10.1186/s13567-019-0630-6

**Published:** 2019-02-18

**Authors:** Jolien Van Cleemput, Katrien C. K. Poelaert, Kathlyn Laval, Hans J. Nauwynck

**Affiliations:** 1 Department of Virology, Parasitology and Immunology, Faculty of Veterinary Medicine, Ghent University, Salisburylaan 133, 9820 Merelbeke, Belgium; 2 Department of Molecular Biology, Princeton University, 119 Lewis Thomas Laboratory, Washington Road, Princeton, NJ 08544 USA

## Abstract

Equine herpesvirus type 5 (EHV5) is a ubiquitous, yet obscure pathogen in the horse population and is commonly associated with fatal equine multinodular pulmonary fibrosis (EMPF). To date, little is known about the precise pathogenesis of EHV5. Here, we evaluated the dynamics of EHV5 infection in representative ex vivo and in vitro equine models, using immunofluorescence staining and virus titration. EHV5 was unable to infect epithelial cells lining the mucosa of nasal and tracheal explants. Similarly, primary equine respiratory epithelial cells (EREC) were not susceptible to EHV5 following inoculation at the apical or basolateral surfaces. Upon direct delivery of EHV5 particles to lung explants, few EHV5-positive cell clusters were observed at 72 hours post-inoculation (hpi). These EHV5-positive cells were identified as cytokeratin-positive alveolar cells. Next, we examined the potential of EHV5 to infect three distinct equine PBMC populations (CD172a^+^ monocytes, CD3^+^ T lymphocytes and Ig light chain^+^ B lymphocytes). Monocytes did not support EHV5 replication. In contrast, up to 10% of inoculated equine T and B lymphocytes synthetized intracellular viral antigens 24 hpi and 72 hpi, respectively. Still, the production of mature virus particles was hampered, as we did not observe an increase in extracellular virus titer. After reaching a peak, the percentage of infected T and B lymphocytes decayed, which was partly due to the onset of apoptosis, but not necrosis. Based on these findings, we propose a model for EHV5 pathogenesis in the horse. Uncovering EHV5 pathogenesis is the corner step to finally contain or even eradicate the virus.

**Electronic supplementary material:**

The online version of this article (10.1186/s13567-019-0630-6) contains supplementary material, which is available to authorized users.

## Introduction

As a member of the *Gammaherpesvirinae* subfamily, equine herpesvirus type 5 (EHV5) is optimally adapted to its natural host, meaning that infected horses are mainly asymptomatic [1]. EHV5 is endemic in the horse population and plenty of horses shed the virus in nasal secretions and/or carry the virus in peripheral blood mononuclear cells (PBMC) or lymphoid organs. Nonetheless, only a small fraction of them develop severe clinical symptoms [2–10]. The virus typically causes upper respiratory tract disease (e.g. pharyngitis) or keratoconjunctivitis accompanied with clinical signs such as nasal and ocular discharge, tachypnea, coughing, fever, enlarged lymph nodes, anorexia, poor body condition and depression [2, 3, 11–13]. Single case reports linked EHV5 to B cell lymphomas, T cell leukemia and dermatitis [14–16]. However, the most dreadful complication of an EHV5 infection is the development of fatal equine multinodular pulmonary fibrosis (EMPF) [17]. EMPF is characterized by the presence of multiple fibrotic nodules throughout the lungs. Histologically, marked interstitial fibrosis with an “alveolar-like” architecture, lined by cuboidal epithelial cells and thickening of the alveolar walls is visible [2, 17, 18]. The high correlation between the presence of EMPF and EHV5 DNA suggests that the virus is involved in the development of lung fibrosis. This is corroborated by the findings of a study on a closely-related gammaherpesvirus murine herpesvirus type 4 (MuHV4). MuHV4 induces lung fibrosis in mice with a progressive deposition of interstitial collagen, increased transforming growth factor β and T helper 2 cytokine expression and hyperplasia of type II pneumocytes [19]. Similarly in humans, the development of idiopathic pulmonary fibrosis has been linked to the gammaherpesvirus Epstein-Barr virus (EBV) [20, 21]. In addition, Williams et al. [22] were able to experimentally induce lung fibrosis in horses upon direct delivery of virulent EHV5 strains into the lungs. However, the choice of viral strain, immunologic status of experimental animals and inoculation route may have favoured the outcome of disease. So far, the exact pathogenic role played by EHV5 in EMPF is unknown. The virus may be an etiologic agent or cofactor in the development of EMPF [2, 22].

Despite the large number of epidemiological studies, little is known about the exact pathogenesis of EHV5 and many statements remain speculative. It is assumed that foals become infected through the upper respiratory tract around the age of 1–6 months [23]. Closely-related gammaherpesviruses, such as human herpesvirus type 8 (HHV8), bovine herpesvirus type 4 (BoHV4) and MuHV4 commonly spread through sexual contact or intrauterine transmission. Still, the presence of EHV5 in the equine reproductive tract has not been reported yet [24–26]. Following primary infection, EHV5 establishes latency to persist in its host. Viral DNA is commonly isolated from blood-derived PBMC (mainly T and B lymphocytes) of healthy horses, indicating that these leukocyte subpopulations are the latency reservoirs of EHV5 [9, 10, 23, 27]. However, the exact mechanism used by EHV5 to reach and infect these cells is unknown. Besides blood- and lymph node-derived PBMC, also alveolar macrophages were found to harbour the virus [2, 17, 18, 22]. However, whether this observation was due to a direct viral infection or a consequence of phagocytosis remains speculative. In the lungs of horses suffering from EMPF, EHV5 antigens were additionally localized in alveolar pneumocytes and interstitial fibroblasts, indicating that the virus can infect these cell types [22].

Although EHV5 is an old pathogen, it only recently attracted the attention of clinicians, horse owners and researchers due to its association with EMPF. Effective therapies are lacking due to the limited knowledge on EHV5 pathogenesis in the horse. Therefore, our study aimed to uncover some of the first key steps herein.

## Materials and methods

### Virus

The equine herpesvirus type 5 (EHV5) KB-P48 strain was kindly provided by Dr K. Borchers and originates from the blood taken of a captive Przewalski’s wild horse [28]. The horse had high immunoperoxidase monolayer assay (IPMA) and virus neutralizing (VN) anti-EHV5 antibody titers, but showed no clinical symptoms. The virus was propagated on rabbit kidney (RK13) cells and used at the 6^th^ passage.

The alphaherpesvirus equine herpesvirus type 1 (EHV1) is known to infect both leukocytes (e.g. CD173a^+^ monocytic cells, T and B lymphocytes) and the respiratory epithelium of the horse [29–31]. Therefore, the EHV1 strain 03P37 was used as a positive control during our viral infection assays. The 03P37 strain originates from the blood taken of a paralytic horse during an outbreak in 2003 [32]. The virus was propagated on RK13 cells and used at the 6^th^ passage.

### Tissue collection and processing

The nasal septa, tracheae and lungs from three different healthy horses were collected at the slaughterhouse. Standardbred and warmblood mares or geldings of 3 to 15 years of age were selected. Tissues were transported in PBS with calcium and magnesium (PBS^+Ca+Mg^), supplemented with 0.1 mg/mL gentamicin (ThermoFisher Scientific, Waltham, MA, USA), 0.1 mg/mL kanamycin (Sigma-Aldrich, St. Louis, MO, USA), 100 U/mL penicillin, 0.1 mg/mL streptomycin (ThermoFisher Scientific) and 0.25 μg/mL amphotericin B (ThermoFisher Scientific).

### Respiratory mucosal explant isolation and cultivation

Nasal and tracheal mucosal explants were prepared and cultivated as previously described [33, 34]. Lung explants were obtained following a technique described for pigs, with minor adaptations [35]. Briefly, lung tissue was first cut up in cubes of approximately 1 cm × 1 cm × 5 cm (W × H × L). These cubes were then transferred to a 20 mL syringe containing 5 mL of 4% agarose (low temperature gelling; Sigma-Aldrich), diluted in PBS. After filling the syringe with 5 mL of additional agarose, it was transferred to 4 °C until the agarose solidified (15 min). The tip of the syringe was cut off, before gently pushing the plunger and thereby moving the embedded lung tissue out of the barrel. Using a cryotome blade, thin lung tissues slices of 1 mm were cut and transferred to a petridish. Here, tissues were thoroughly washed to remove excess agarose and finally trimmed to a surface of approximately 25 mm^2^. Lung explants were transferred to 6-well plates, submerged in serum-free medium (DMEM/RPMI [ThermoFisher Scientific], supplemented with 0.1 mg/mL gentamicin, 100 U/mL penicillin, 0.1 mg/mL streptomycin, and 0.25 μg/mL amphotericin B) and cultivated at 37 °C and 5% CO_2_.

### EREC isolation and cultivation

Primary equine respiratory epithelial cells (EREC) were isolated and cultured as described previously [34, 36].

### Isolation of equine monocytes, T and B lymphocytes

Equine PBMC were isolated as described previously [31]. The collection of blood was approved by the ethical committee of Ghent University (EC2017/118). Ten hours post seeding, CD172a^+^ monocytic cells had adhered to the plastic (purity > 90%, as assessed by flow cytometry [31]) and non-adherent cells consisted of two dominant leukocyte populations: T and B lymphocytes. Following removal of non-adherent cells, equine CD172a^+^ monocytes were further maintained in RPMI supplemented with 5% fetal calf serum (FCS) and antibiotics. Equine T lymphocytes were separated from B lymphocytes by negative selection magnetic-activated cell sorting (MACS). In summary, 5 × 10^7^ cells were incubated with a mouse anti-horse pan B lymphocyte antibody (clone CVS36, directed against the equine Ig light chains; Bio-Rad, Oxford, UK), diluted in PBS with 10% negative goat serum (NGS) for 1 h at 4 °C. Cells were washed in ice-cold elution buffer (PBS + 2 mM EDTA + 2% FCS) and re-suspended in elution buffer, containing 100 µL rat anti-mouse IgG micro-beads (MACS Miltenyi Biotec, Cologne, Germany) for 1 h at 4 °C. Next, cells were washed in elution buffer before transferring them onto a LS column (MACS Miltenyi Biotec). The cell-fraction that went through the column was collected and contained over 95% positive CD3^+^ T lymphocytes, as assessed by flow cytometry after indirect immunofluorescence staining with a mouse anti-equine CD3 monoclonal antibody (clone UC_F6G; California University, Davis, Additional file 1A). The remaining cell-fraction contained the Ig light chain^+^ B lymphocytes, as assessed by flow cytometry after indirect immunofluorescence staining with a mouse anti-pan B lymphocyte antibody (clone CVS36; Additional file 1B). Finally, equine T and B lymphocytes were counted using a Bürker counting chamber and seeded in RPMI supplemented with 5% FCS, 1% MEM non-essential amino-acids, 1% sodium pyruvate, 4 U/mL interleukin-2 and antibiotics.

### Viral infection assays

#### Respiratory mucosal explants

Explants were cultured 24 h for adaptation before thoroughly washing and transferring them to a clean 24-well plate, as previously published [33]. While lung explants were left untreated, nasal and tracheal explants were incubated with 8 mM EGTA or PBS^+Ca+Mg^ (control) for 1 h at 37 °C to dissociate intercellular junctions [34]. Following a thorough washing step, nasal, tracheal and lung explants were subsequently exposed to medium alone (mock), the KB-P48 EHV5 strain (10^6.5^ TCID_50_) or the 03P37 EHV1 strain (10^6.5^ TCID_50_, positive control) for 1 h at 37 °C. Explants were then washed 3 times in PBS to remove unbound virus particles. Finally, explants were placed back onto their gauzes and serum-free medium was added. At corresponding time points, explants were placed in methylcellulose-filled plastic tubes and snap-frozen at −80 °C until further processing.

#### EREC

We recently described a protocol for apical versus basolateral infection of EREC by EHV1 in a transwell system [34]. Cells were grown to confluency and the trans-epithelial electrical resistance (TEER) was measured daily until a steady TEER of ~ 500–700 Ω × cm^−2^ was attained. The apical surface of EREC was then treated with 8 mM EGTA or PBS^+Ca+Mg^ for 30 min at 37 °C to dissociate the intercellular junctions. Following a washing step in PBS, cells were exposed to medium alone (mock), 100 μL KB-P48 EHV5 strain (MOI of 1) or 03P37 EHV1 strain (MOI of 1) at either the apical or the inverted basolateral surface for 1 h at 37 °C. Non-adsorbed virus particles were removed by washing the EREC three times with DMEM/F12. Fresh EREC-medium was added to the platewells and cells were further incubated at the air–liquid interface. At corresponding time points, cells were fixed in methanol for 20 min at −20 °C and stored dry at −20 °C until further processing.

#### Equine monocytes, T and B lymphocytes

Monocytes, grown on cover slips, were mock-inoculated or inoculated with either EHV5 (MOI 1 or 10) or EHV1 (MOI 1; positive control) in 200 µL monocyte medium for 1 h at 37 °C. Afterwards, the cells were gently washed twice to remove the inoculum and further incubated with fresh medium. At 6, 24, 48, 72 and 96 hpi (hours post-inoculation), cell supernatant was collected, and cells were fixed in methanol for 20 min at −20 °C and stored dry at −20 °C until further processing.

T and B lymphocytes were inoculated at a concentration of 2.5 × 10^6^ cells/mL with EHV5 (MOI 1 or 10) or EHV1 (MOI 1) diluted in lymphocyte medium for 1 h at 37 °C. The inoculum was removed by 2 centrifugation steps at 300 × *g* and cells were further incubated in 24-well plates with fresh medium. At 6, 24, 48, 72 and 96 hpi, cells were pelleted by centrifugation at 300 *g*. The supernatant, containing free virus particles, was collected and cells were fixed in 1% paraformaldehyde (PFA) for 10 min at room temperature (RT) and finally stored in PBS at 4 °C until further processing.

#### Polyclonal anti-EHV5 antibody

The polyclonal horse anti-EHV5 antibody originates from blood taken of a Shetland pony stallion (Sultan) intended for routine diagnostic serological examination. The pony was kept in a premise, where one of the five horses (Haflinger breed) showed signs of nasal discharge and dullness and was diagnosed with EHV5 by PCR on a nasal swab. The affected horse was isolated from the herd and all horses (the Haflinger and four Shetland ponies) were screened for the presence of EHV5-specific antibodies, regarding further isolation management. The amount of anti-EHV5 specific IPMA and VN antibodies was semi-quantitatively determined on RK13 cells using an IPMA (10^2^ TCID_50_ KB-P48) or a seroneutralization test, respectively. Antibodies against EHV5 were present in the sera of all five horses and the titer ranged from 2 to > 256 (Sultan) for VN antibodies and from 2560 to 40,960 (Sultan) for IPMA antibodies. The antibodies from Sultan’s serum were then purified and biotinylated, similarly to the polyclonal horse anti-EHV1 antibody previously made in our lab [37]. As shown in Additional file 2, left panels, the positive signal in EHV5-infected RK13 cells following both immunofluorescence (A) and immunocytological (B) staining with the biotinylated Sultan antibody (1:20) and subsequent incubation with streptavidin-FITC^®^ or streptavidin-HRP, respectively, confirmed its suitability in further staining experiments. The biotinylated polyclonal horse anti-EHV1 antibody mixture did not contain anti-EHV5 antibodies and was used as a negative control (Additional file 2, right panels).

### Immunofluorescence staining and confocal microscopy

#### Explants

Sixteen μm thick cryosections of equine nasal, tracheal and lung explants were cut using a cryostat at −20 °C and loaded onto 3-aminopropyltriethoxysilane-coated (Sigma-Aldrich) glass slides. Slides were then fixed in 4% PFA for 15 min and subsequently permeabilized in 0.1% Triton-X 100 diluted in PBS. Non-specific binding sites (e.g. equine IgG receptor) were first blocked by 45 min incubation with 10% negative horse serum, obtained during a previous in vivo study [38], diluted in PBS at 37 °C. To label EHV5 and EHV1 proteins, the polyclonal biotinylated horse anti-EHV5 antibody (Sultan; 1:20) or polyclonal biotinylated horse anti-EHV1 antibody, respectively, was used for 1 h at 37 °C, followed by incubation with streptavidin-FITC^®^ for 1 h at 37 °C. Nuclei were detected by staining with Hoechst 33342 (ThermoFisher Scientific). Transwell membranes were excised from the culture inserts and mounted on glass slides using glycerol-DABCO. The number of viral plaques and/or single infected cells was evaluated on 100 consecutive cryosections, using confocal microscopy.

A double immunofluorescence staining of lung explant cryosections was performed to identify EHV5-positive cells as cytokeratin-positive. For this, cryosections were incubated for 1 h with the polyclonal biotinylated anti-EHV5 antibody (1:20), together with the monoclonal mouse anti-pan cytokeratin antibody (clone AE1/AE3; Agilent, Santa Clara, USA; 1:100). After a washing step, cryosections were incubated with streptavidin-FITC^®^ and a goat anti-mouse IgG Texas Red^®^-conjugated antibody (ThermoFisher Scientific). Nuclei were detected by staining with Hoechst 33342.

#### EREC

Methanol-fixed EREC were directly stained in the transwells, as described above. The complete EREC monolayer was analysed using a Leica (TCS SPE) confocal microscope. As a negative control, mock-inoculated cells were stained following the above protocols. The polyclonal horse anti-EHV1 antibody was included as isotype control antibody [37].

#### Equine monocytes, T and B lymphocytes

Methanol-fixed monocytes, grown on cover slips, were stained directly in the wells. PFA-fixed lymphocytes were cytospinned onto 3-aminopropyltriethoxysilane-coated (Sigma-Aldrich) glass slides and subsequently permeabilized in 0.1% Triton-X 100 diluted in PBS. Immunofluorescence staining further proceeded as described previously. Slides were mounted with glycerol-DABCO and analysed using confocal microscopy. The percentage of viral antigen-positive cells was calculated based on 300 cells counted in 5 distinct fields. In EHV5-infected lymphocytes, the percentage of cells showing DNA fragmentation due to EHV5 infection was additionally determined.

#### Cell death analysis

The percentage of mock- or EHV5-inoculated cells showing signs of apoptosis (annexin V-positive) or necrosis (propidium iodide positive) was determined 72 hpi, using the “Dead Cell Apoptosis Kit” from ThermoFisher Scientific (V13241). Live cells were incubated with the appropriate reagents following the manufacturer’s guidelines. Next, cells were fixed in 1% PFA and stained for EHV5 antigens, as described above. The percentage of apoptotic or necrotic cells was calculated based on 300 cells counted in 5 distinct fields using confocal microscopy.

### Virus titration

Cell and explant supernatants were collected at various time points and stored at −80 °C until titration. EHV1 and EHV5 titrations were conducted on RK13 cells, which were incubated at 37 °C for 7 days. EHV1 titers were determined based on cytopathogenic effect. EHV5 titers were determined based on EHV5-immunocytological staining. Briefly, RK13 cells were washed in PBS, air-dried at 37 °C for 1 h and frozen at −20 °C for a minimum of 2 h. After thawing, cells were fixed in 4% PFA at 4 °C for 15 min. Non-specific binding sites were blocked by incubating the cells with a mixture of tris-buffered saline (TBS), supplemented with 5% NGS for 20 min at 37 °C. EHV5-positive cells were stained with the anti-EHV5 serum (Sultan), diluted 1:1000 in TBS with 2% NGS. After washing, a goat anti-horse IgG-peroxidase (Jackson ImmunoResearch, Cambridgeshire, UK) was added in TBS. For detection, a 5% aminoethyl carbazole (AEC) solution, supplemented with 0.025% H_2_O_2_ was added to the wells for 10 min at 37 °C. The enzymatic reaction was stopped by washing the cells in PBS. All titers were expressed as TCID_50_.

### Statistical analyses

Significant differences (*P* < 0.05) between different time points or different MOI were identified by analysis of variances (ANOVA) followed by Tukey’s post hoc test. If homoscedasticity of the variables was not met as assessed by Levene’s test, the data were log-transformed prior to ANOVA. Normality of the residuals was verified by the use of the Shapiro–Wilk test. If the variables remained heteroscedastic or normality was not met after log-transformation, a Kruskal–Wallis’ test, followed by a Mann–Whitney’s post hoc test were performed. Significant differences in the percentage of apoptotic cells between mock or EHV5 inoculations were identified by a Student’s *t* test. All analyses were conducted in IBM SPSS Statistics for Windows, version 25.0 (IBM Corp, Armonck, NY, USA).

## Results

### EHV5 infects lung alveolar cells but not the equine ciliated respiratory epithelium lining the nasal septum and trachea

To date, it is unclear how exactly EHV5 establishes a life-long infection in new hosts. The virus is delivered to the respiratory tract through inhalation and somehow finds its way to latency reservoirs (PBMC). Here, we examined whether EHV5 primarily infects equine respiratory epithelial cells using nasal and tracheal mucosal explant models and primary EREC. In addition, we examined whether EHV5 is able to infect cells within lung explants upon direct delivery.

#### Explants

Over the time course of the experiment (24, 48 and 72 hpi), EHV5-infected cells were not detected in the respiratory epithelium of nasal and tracheal mucosal explants. In contrast, at 24 hpi, we counted an average of 3 ± 3 and 32 ± 15 EHV1 plaques in 8 mm^2^ respiratory epithelium of nasal and tracheal mucosal explants, respectively. As EHV1 infection is known to be enhanced upon disruption of epithelial integrity [34], nasal and tracheal mucosal explants were treated with EGTA prior to inoculation with EHV5. Despite the EGTA treatment, EHV5-infected cells were not found in the respiratory epithelium of these explants. Finally, EHV1-positive leukocytes were observed beneath the basement membrane 24 hpi. However, EHV5-positive leukocytes were absent in the EHV5-inoculated nasal and tracheal mucosal explants at all time points.

**Figure 1:**
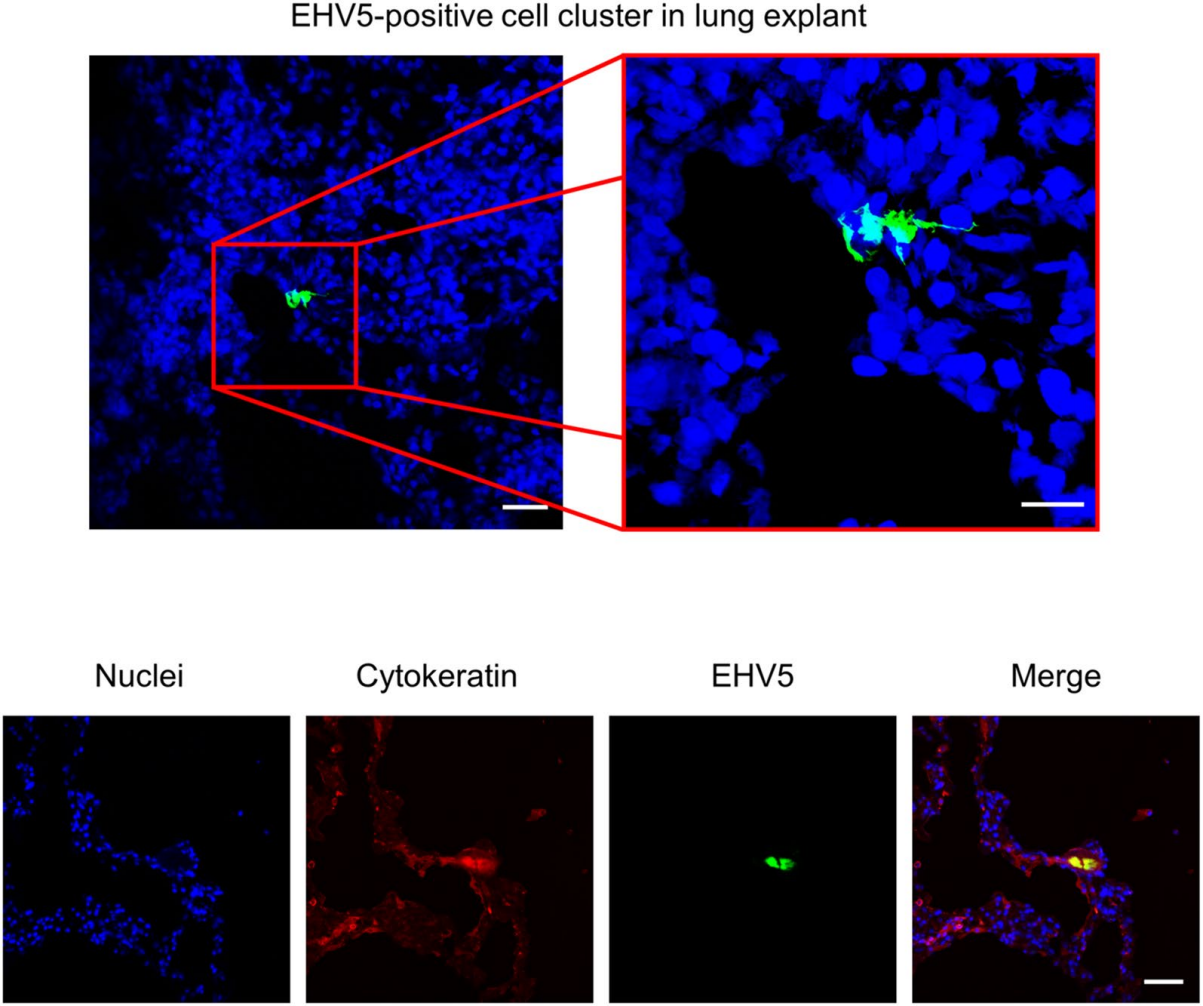
**EHV5 antigen expression 72 hpi in localized cell clusters within EHV5-inoculated lung explants.** In the upper panel, cryosections were stained for EHV5 antigens (Sultan; green) and cell nuclei (Hoechst 33342; blue). In the lower panel, cryosections were simultaneously stained for EHV5 antigens (Sultan; green), cytokeratin (AE1/AE3; red) and cell nuclei (Hoechst 33342; blue). The scale bars represent 50 µm.

In lung explants, however, a small amount (22 ± 9) of EHV5-infected cells was present in a volume of 8 mm^3^ lung tissue at 72 hpi. EHV5-positive cells were usually found in a cell cluster of approximately 4 ± 2 EHV5-positive cells per cluster. Double immunofluorescence staining for EHV5 antigens and cytokeratin confirmed that these infected cell clusters were of epithelial origins. Representative confocal images are given in Figure 1.

Nasal, tracheal and lung explants did not produce detectable progeny EHV5 particles 72 hpi, as viral titers in the supernatant remained below 1 log_10_TCID_50_/mL. The supernatant of nasal and tracheal mucosal explants 24 hpi contained an EHV1 titer of 2.4 ± 0.9 and 2.5 ± 0.5 log_10_TCID_50_/mL, respectively.

#### EREC

EHV1 formed 1 ± 1 and 55 ± 26 viral plaques in 3 × 10^4^ EREC 10 hpi following inoculation at the apical or basolateral surfaces, respectively. In contrast, none of the cells were EHV5-positive 96 h following inoculation at both surfaces. Similarly as observed in nasal and tracheal explants, disruption of EREC integrity with EGTA prior to inoculation did not overcome the restriction to EHV5 infection of the cells.

### EHV5 does not replicate in equine monocytic cells but induces a lytic infection in equine T and B lymphocytes in vitro

As the equine ciliated respiratory epithelium did not support EHV5 growth, we hypothesized that EHV5 directly infects PBMC. In comparison, the human gammaherpesvirus Epstein-Barr virus (EBV) is able to synthetize viral proteins and produce a lytic infection in human B lymphoblasts [39]. Therefore, we examined the ability of EHV5 to infect and replicate in equine PBMC. The kinetics of viral protein expression and virus production in equine CD3^+^ T lymphocytes, Ig light chain^+^ B lymphocytes and CD172a^+^ monocytes was evaluated by confocal microscopy and virus titration on cell supernatant, respectively. Parallel mock inoculations confirmed the absence of EHV5-positive T lymphocytes, B lymphocytes and monocytes in the blood donor-derived PBMC.

#### T lymphocytes

**Figure 2:**
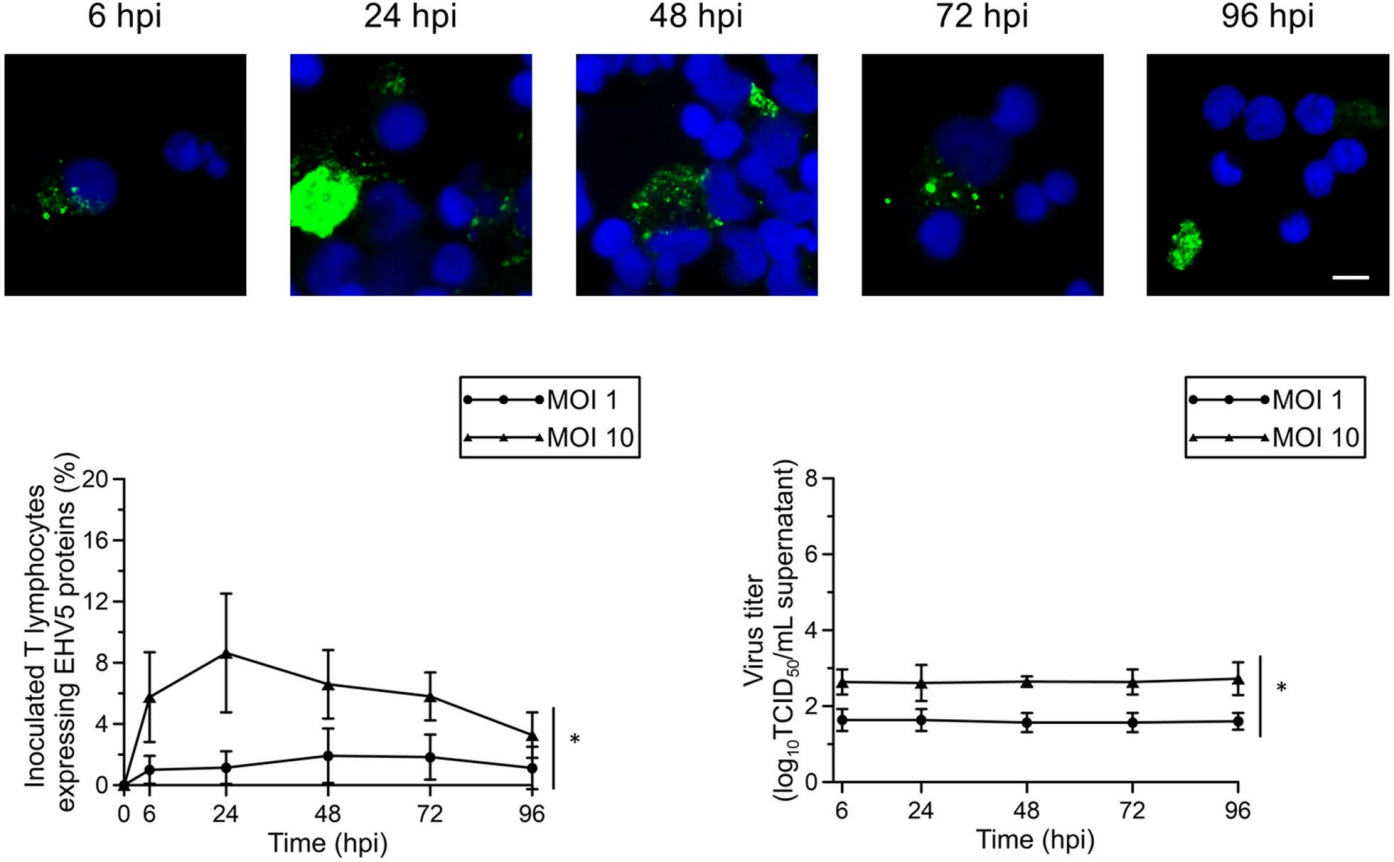
**Expression of EHV5 antigens in EHV5-inoculated (MOI 1 or 10) T lymphocytes.** At indicated time points, supernatant was collected for virus titration and cells were fixed and immunofluorescently stained for EHV5 antigens. Upper panel; representative confocal images of EHV5 antigen expression (Sultan; green) in T lymphocytes. Cell nuclei were counterstained with Hoechst 33342 (blue). The scale bar represents 5 µm. Lower left panel; the percentage of EHV5-positive cells was calculated based on 300 cells counted in 5 distinct field. Lower right panel; the virus titer was determined in supernatant on RK13 cells. Data are represented as mean ± SD and asterisks indicate significant differences (*P < 0.05) between MOI 1 and 10. Experiments were performed on cells from 3 individual horses.

In EHV5-inoculated T lymphocytes (MOI of 1), 1 ± 1% of the cells started to express viral proteins in the cytoplasm at 6 hpi, as shown in Figure 2, left graph. This percentage slightly, but not significantly increased over time to 2 ± 2% at 48 hpi and declined again to 1 ± 1% at 96 hpi. Increasing the MOI 10 times rapidly and significantly (*P* < 0.05) increased the percentage of infected cells to 6 ± 3% at 6 hpi and 9 ± 4% at 24 hpi. Starting from this time point, the percentage of EHV5-infected T lymphocytes declined gradually to 3 ± 1.5% at 96 hpi. Representative confocal images are shown in the upper panel of Figure 2. EHV1 antigens were visible in 1 ± 0.3% equine T lymphocytes 24 hpi.

No significant increase in extracellular EHV5 titer was observed over the course of the experiment (Figure 2, right graph).

#### B lymphocytes

**Figure 3:**
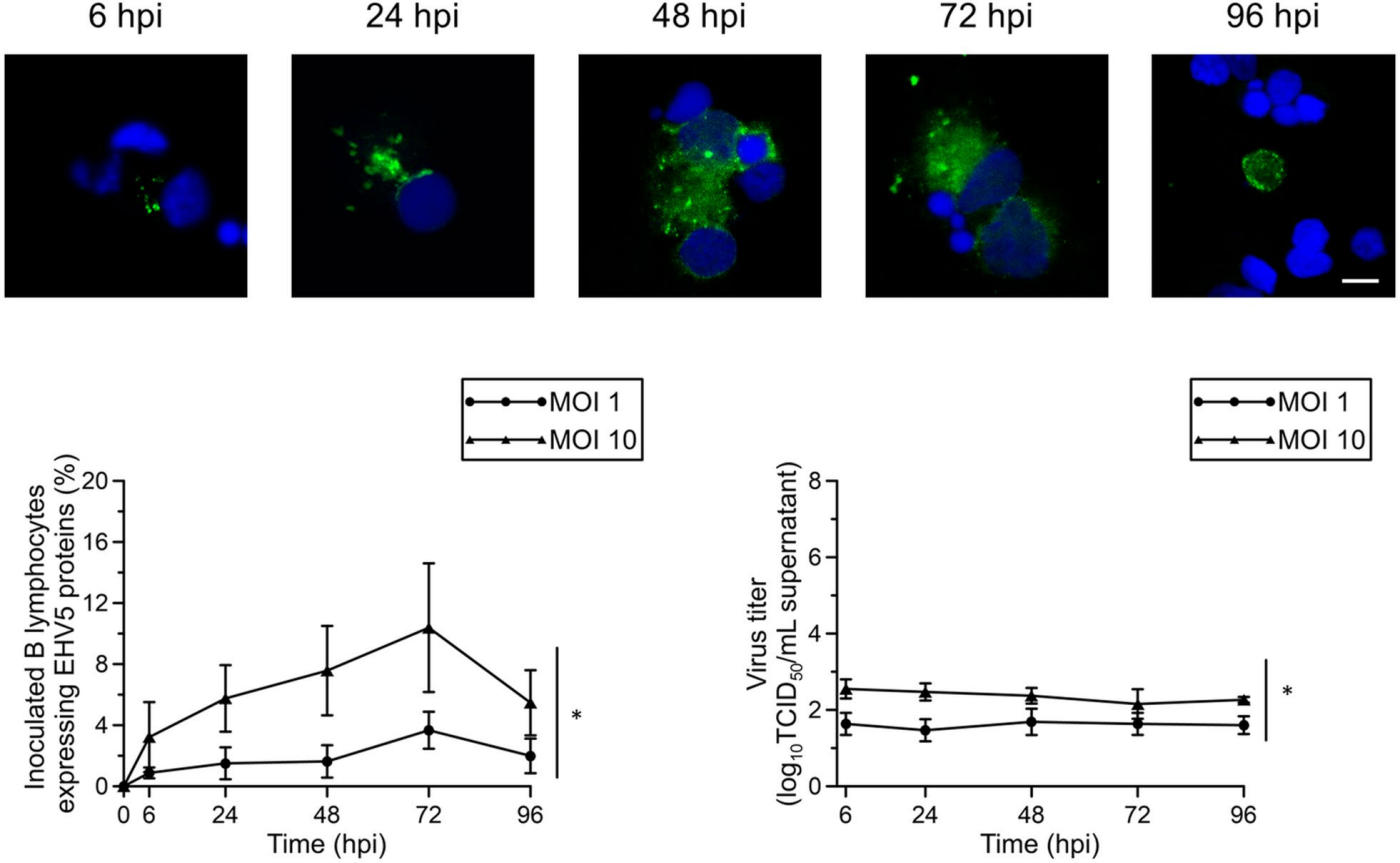
**Expression of EHV5 antigens in EHV5-inoculated (MOI 1 or 10) B lymphocytes.** At indicated time points, supernatant was collected for virus titration and cells were fixed and immunofluorescently stained for EHV5 antigens. Representative confocal images of EHV5 antigen expression (Sultan; green) in B lymphocytes. Cell nuclei were counterstained with Hoechst 33342 (blue). The scale bar represents 5 µm (upper panel). The percentage of EHV5-positive cells was calculated based on 300 cells counted in 5 distinct fields (lower left panel). The virus titer was determined in supernatant on RK13 cells (lower right panel). Data are represented as mean ± SD and asterisks indicate significant differences (*P < 0.05) between MOI 1 and 10. Experiments were performed on cells from 3 individual horses.

EHV5 inoculation of B lymphocytes at a MOI of 1 resulted in an average of 1 ± 0.5% EHV5-positive cells 6 hpi (Figure 3, left graph). This percentage increased over time to a peak of 3.5 ± 1% at 72 hpi, which was significantly (*P* < 0.05) different from the percentages at 6, 24 and 48 hpi. At 96 hpi, only 2 ± 1% of the inoculated B lymphocytes remained EHV5-positive. Again, increasing the MOI to 10 resulted in a significant (*P* < 0.05) increase in cells expressing EHV5 proteins already at 6 hpi (3 ± 2%). This percentage further increased in a time-dependent manner to 10 ± 4% at 72 hpi. Similarly as to EHV5-inoculated T lymphocytes, the percentage of EHV5-positive inoculated B lymphocytes decreased again at 96 hpi (5.5 ± 2%). Representative confocal images are shown in the upper panel of Figure 3. We observed 0.5 ± 0.2% EHV1-positive equine B lymphocytes 24 hpi.

No significant increase in extracellular EHV5 titer was observed over the course of the experiment (Figure 3, right graph).

#### Monocytes

EHV5 proteins were not detected in EHV5-inoculated equine monocytes throughout the course of the experiment. In contrast, EHV1 antigens were expressed in 3.7 ± 1.4% of the inoculated monocytes 24 hpi.

### EHV5 lytic infection causes nuclear fragmentation and apoptosis in equine T and B lymphocytes

It is known that EBV induces DNA fragmentation during lytic infection of human B lymphoblasts [39]. This DNA fragmentation contributes to the cytopathic effect of EBV and eventually ends in cell death. As EHV5 was able to induce a lytic replication in equine T and B lymphocytes, we analysed whether cell nuclear morphology changed upon infection using Hoechst 33342. The fluorescent dye Hoechst 33342 binds to the minor groove of double-stranded DNA and can be used in immunofluorescence staining to identify chromatin condensation and nuclear fragmentation [40]. As nuclear fragmentation preludes cell death, we additionally analysed the percentage of cells showing signs of apoptosis (annexin V-positive) or necrosis (propidium iodide-positive) [41]. Apoptosis is a tightly regulated form of cell death and can be recognized by the binding of annexin V to phosphatidyl serine on the cell surface [42]. In contrast, propridium iodide can penetrate the plasma membrane of necrotic cells and subsequently binds to nucleic acids.

#### Nuclear fragmentation

**Figure 4:**
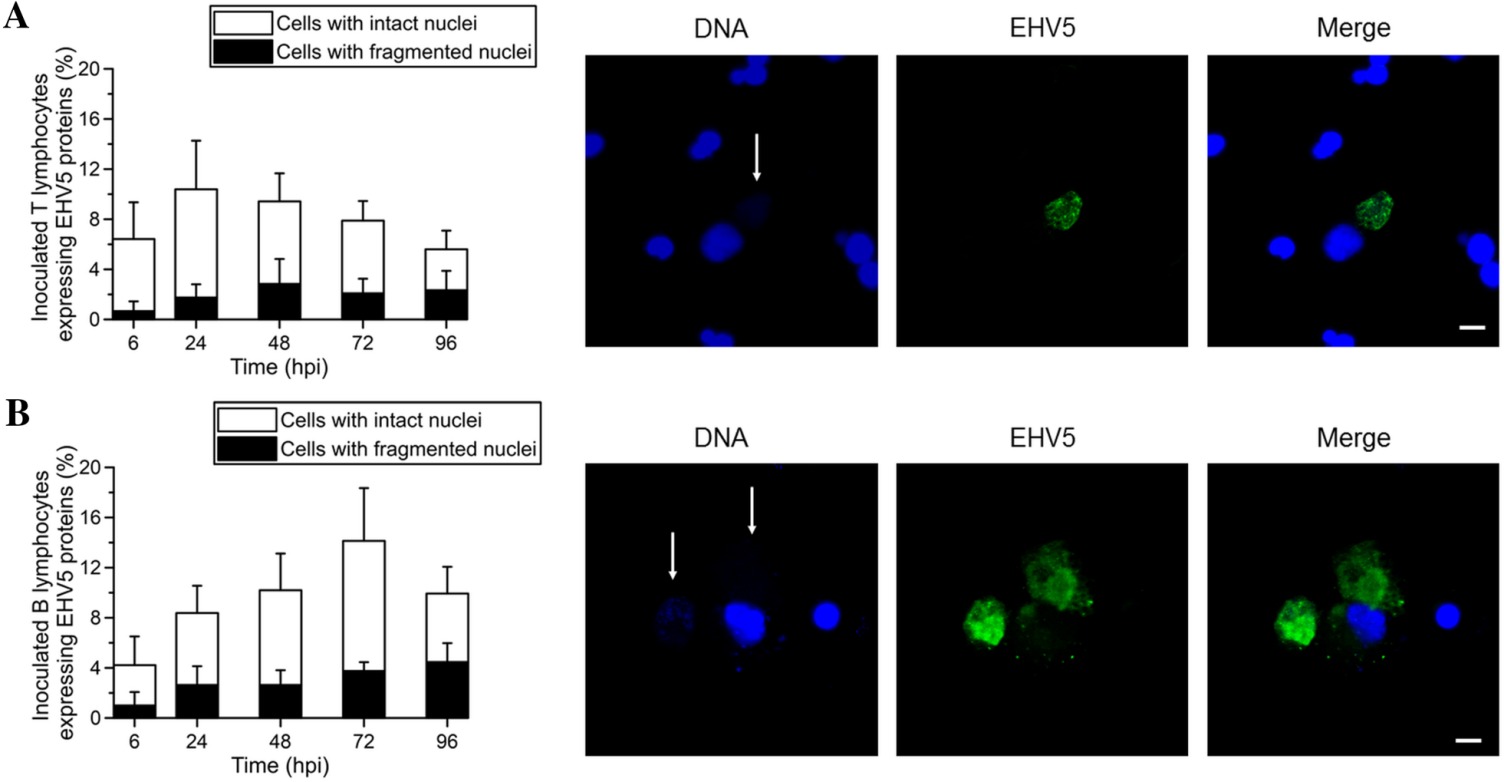
**Induction of chromatin condensation and nuclear fragmentation in EHV5 antigen-expressing T lymphocytes (A) and B lymphocytes (B) following inoculation at a MOI of 10.** Left panels; the percentage of EHV5-inoculated cells expressing both EHV5 antigens and nuclear fragmentation is indicated by black bars. White bars represent the percentage of inoculated cells that express EHV5 antigens, but show no signs of nuclear fragmentation. Data are represented as mean + SD and were obtained from 3 individual horses. Right panels; representative confocal images of EHV5 expression (Sultan; green) in T and B lymphocytes. Cell nuclei were counterstained with Hoechst 33342 (blue). Note the signs of nuclear fragmentation (i.e. translucent and/or compartmented appearance of cell nuclei, as shown by the translucent and/or punctuated Hoechst signal; white arrows). The scale bars represent 5 µm.

Starting from 6 hpi, we observed that EHV5 viral proteins co-localized with the nucleus of approximately 0.5 ± 0.5% of the EHV5-inoculated (MOI 10) T lymphocytes (Figure 4A) and 1 ± 1% of EHV5-inoculated (MOI 10) B lymphocytes (Figure 4B). Interestingly, all of these cells exhibited a translucent and/or punctuated Hoechst signal, as shown in the right panels of Figure 4A and B. The number of EHV5-positive T lymphocytes showing signs of nuclear fragmentation significantly (*P* < 0.05) increased to 3 ± 1% at 48 hpi and remained stable starting from this time point. The number of EHV5-positive B lymphocytes showing signs of nuclear fragmentation steadily increased in a time-dependent manner to 4.5 ± 1.5% at 96 hpi. In contrast, less than 1% of mock-inoculated cells showed signs of nuclear fragmentation throughout the course of the experiment.

#### Cell death analysis

**Figure 5:**
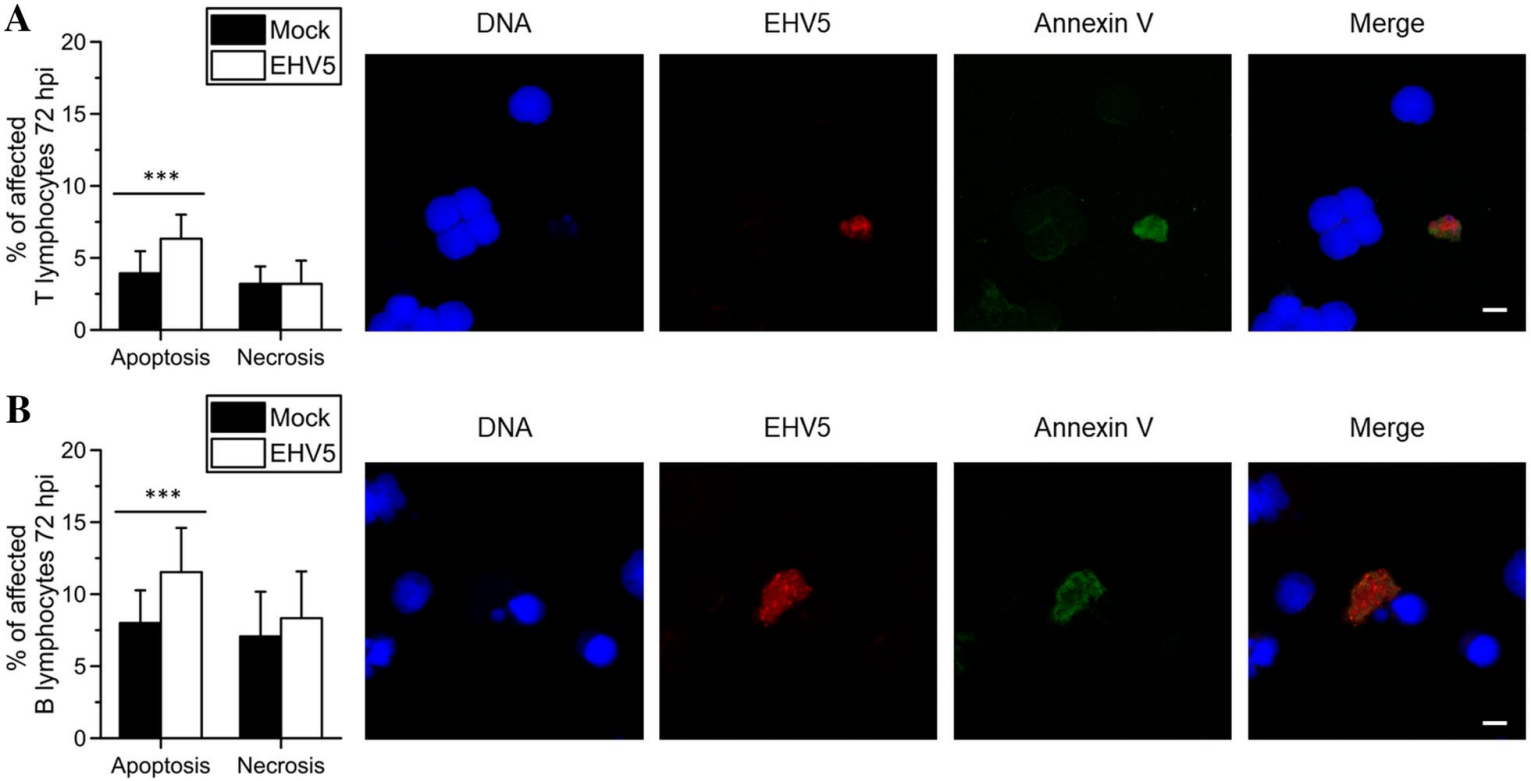
**Induction of apoptosis in EHV5 antigen-expressing T lymphocytes (A) and B lymphocytes (B) following inoculation at a MOI of 10 (72 hpi).** Left panels; the percentage of mock- (black bars) or EHV5- (white bars) inoculated cells showing signs of apoptosis (left) or necrosis (right). Data are represented as means + SD and asterisks indicate significant differences (***P < 0.001) between mock- and EHV5-inoculated cells. Experiments were performed on cells from 3 individual horses. Cell death was analysed on living cells by the “Dead Cell Apoptosis Kit” from ThermoFisher Scientific. Apoptosis was characterized by the binding of annexin V to cellular phosphatidyl serine and necrosis was identified by binding of propidium iodide to nucleic acids in the cell. Right panels; after incubation with annexin V-FITC^®^ (green), cells were fixed and stained for EHV5 antigens (Sultan; red). Cell nuclei are counterstained in blue. The scale bars represent 5 µm.

As shown in Figure 5, the percentage of apoptotic cells was significantly (*P* < 0.001) higher in EHV5-inoculated T lymphocytes (6.5 ± 1.5%) and B lymphocytes (11.5 ± 3%) 72 hpi, compared to mock-inoculated T lymphocytes (4 ± 1.5%) and B lymphocytes (8 ± 2%), respectively. Simultaneously staining apoptosis (annexin V) and EHV5 antigens confirmed their co-localisation in both T and B lymphocytes, as illustrated in the right panels of Figure 5.

No significant difference was found in the percentage of necrotic cells between EHV5- and mock-inoculated T and B lymphocytes at 72 hpi.

## Discussion

The present study aimed at uncovering some of the first crucial steps in EHV5 pathogenesis, starting with the identification of susceptible target cells. For this, we first examined whether EHV5 can replicate in epithelial cells lining the horse’s respiratory tract. Next, we evaluated EHV5 replication kinetics in different PBMC subpopulations, as PBMC are the presumable viral latency reservoirs.

Following direct delivery of EHV5 to equine nasal septum or tracheal mucosal explants, viral protein expression was not detected in respiratory epithelial cells or in single patrolling immune cells. These findings were corroborated in primary equine respiratory epithelial cells (EREC), in which no EHV5-positive cells were found following inoculation at both the apical or basolateral surface. In comparison, human epithelial cells are difficult to infect in vitro with the human gammaherpesvirus Epstein–Barr virus (EBV) [43]. However, the virus is able to efficiently infect epithelial cells following EBV propagation in B lymphocytes [44]. On the contrary, epithelial-cell derived virus particles can infect B lymphocytes more efficiently. This state-of-the-art alternating cell tropism is facilitated through the degradation of viral gp42 by MHC II trafficking in B lymphocytes, thereby liberating gH/gL from the gp42/gH/gL complex. It was proposed that free gH/gL complexes are necessary for the interaction between the virion and epithelial cells. These observations may be in line with our data, as the epithelial cell-derived stock of EHV5 could efficiently infect equine T and B lymphocytes, but was unable to infect the ciliated respiratory epithelium. Interestingly, a few alveolar cells became EHV5-positive following inoculation of equine lung explants. In an in vivo study from Williams et al. [22], EHV5 antigens were also detected in alveolar epithelial cells upon direct delivery of virus particles to the lungs. In addition, several studies already reported the presence of EHV5 DNA in equine lung tissues [45–47]. However, care must be taken by extrapolating these results to the real in vivo situation, as it is highly unlikely that free virus particles can directly access the lungs upon inhalation in healthy horses. Indeed, most viruses that overcome the nasal filter end up in the trachea and are disposed by the mucociliary escalator [48, 49].

Next, we demonstrated that the percentage of EHV5-positive T and B lymphocytes increased over time upon in vitro inoculation, reaching a peak at 24 hpi and 72 hpi, respectively, and then declined. This decay might indicate that infection was cleared. For example, apoptosis or controlled cell destruction can act as an innate response to counteract viral infection. Indeed, apoptosis prevents viral dissemination, as the cell is carefully disassembled and cleared by the host’s immune system [50]. On the contrary, uncontrolled necrosis is unfavourable for the host, as this results in the release of cytoplasmic material, including viral particles. In turn, these viral particles might spread in the host and infect new cells. Here, apoptosis, but not necrosis, was induced in up to 50% of the infected equine T and B lymphocytes. In comparison, EBV early proteins participate in the fragmentation of chromosomal DNA and the onset of apoptosis during lytic infection of human lymphoblasts in vitro [39]. However, the high MOI used in our and the latter experiment might have favoured the onset of apoptosis. Transcription and translation of a high number of viral DNA copies might have flooded the cellular endoplasmic reticulum with viral proteins destined for assembly. In turn, overload of the endoplasmic reticulum could have elicited a cascade of signal transduction pathways, eventually leading to apoptosis [50]. Indeed, it seems unlikely that a virus, so optimally adapted to its host, kills its host cell on purpose. On the contrary, multiple gammaherpesviruses (e.g. EBV, HHV8, BoHV4) have evolved mechanisms to induce latency and inhibit apoptosis to prolong their survival in the host [51–54]. For example in latently EBV-infected B lymphocytes, only a limited number of viral-coded proteins are expressed, including latent membrane protein 1 (LMP1). As this protein interacts with apoptotic signals, the virus cleverly guides the infected B lymphocyte towards a long-living (memory) state [55]. The establishment of EHV5 latency in equine T and B lymphocytes could further explain the drop of EHV5-positive cells starting from 24 and 72 hpi, respectively.

Although infected lymphocytes clearly produced viral proteins intracellularly, the extracellular virus titer did not increase throughout the course of the experiment. The low viral titers that were observed at all time points presumably reflect remnant inoculum viral particles. In dying cells, viral proteins were contained through apoptosis. In live EHV5-infected cells, however, assembly of virus particles and/or release of cell-free progeny virions into surroundings must have been hampered. Indeed, herpesvirus infections are commonly non-productive in leukocytes and this strategy allows the virus to remain in its host, undetectable by the immune system [31, 56, 57]. Still, we frequently observed clustering of EHV5-positive T or B lymphocytes, indicating that the virus may spread via cell–cell transfer. Cell-to-cell transfer is a well-known strategy used by herpesviruses to bypass the hostile immune environment of the host, containing phagocytes, antibodies and complement [58, 59]. Indeed, previous studies demonstrated that the efficiency of EBV transfer from B lymphocytes to epithelial cells was highly upregulated by cell–cell contact [58, 60]. Binding of EBV gp350 with the B lymphocyte surface protein CD21 was proposed to unmask other putative viral glycoproteins, essential for epithelial cell binding. To investigate whether EHV5 could also be transferred from lymphocytes to EREC, we co-cultured infected lymphocytes at the apical surface of naïve EREC. Still, EHV5 was unable to infect and replicate in EREC (data not shown). It would be interesting to assess viral transfer from lymphocytes to the basolateral surface of EREC, as it would be the case in vivo. For example, EBV transfer infection of polarized epithelial cells is restricted to the basolateral surface, even though cell–cell contacts are also established at the apical surface [61]. The researchers suggested that putative EBV binding and entry receptors on the epithelial cells are similarly restricted to the basolateral surface. Unfortunately, we could not perform this experiment due to technical limitations. The small pore size of the transwells, necessary for EREC support, did not allow sufficient cell–cell contacts between the basolateral surface of EREC and equine lymphocytes (data not shown).

In our study, EHV5 did not replicate in equine monocytes in vitro and in fibroblasts of ex vivo mucosal explants. This is in contrast with a study from Williams et al. [22], who found viral antigens in the alveolar macrophages and interstitial fibroblasts of the lungs in vivo. Differentiated macrophages are more specialized for phagocytosis than monocytes. Thus, the presence of viral antigens within alveolar macrophages of infected horses could merely be a consequence of phagocytosis. In addition, EHV5-positive fibroblasts were only found in a limited number of infected horses several weeks following initial challenge. In our short-living ex vivo explant system, EHV5 might not have been able to infect fibroblasts.

**Figure 6:**
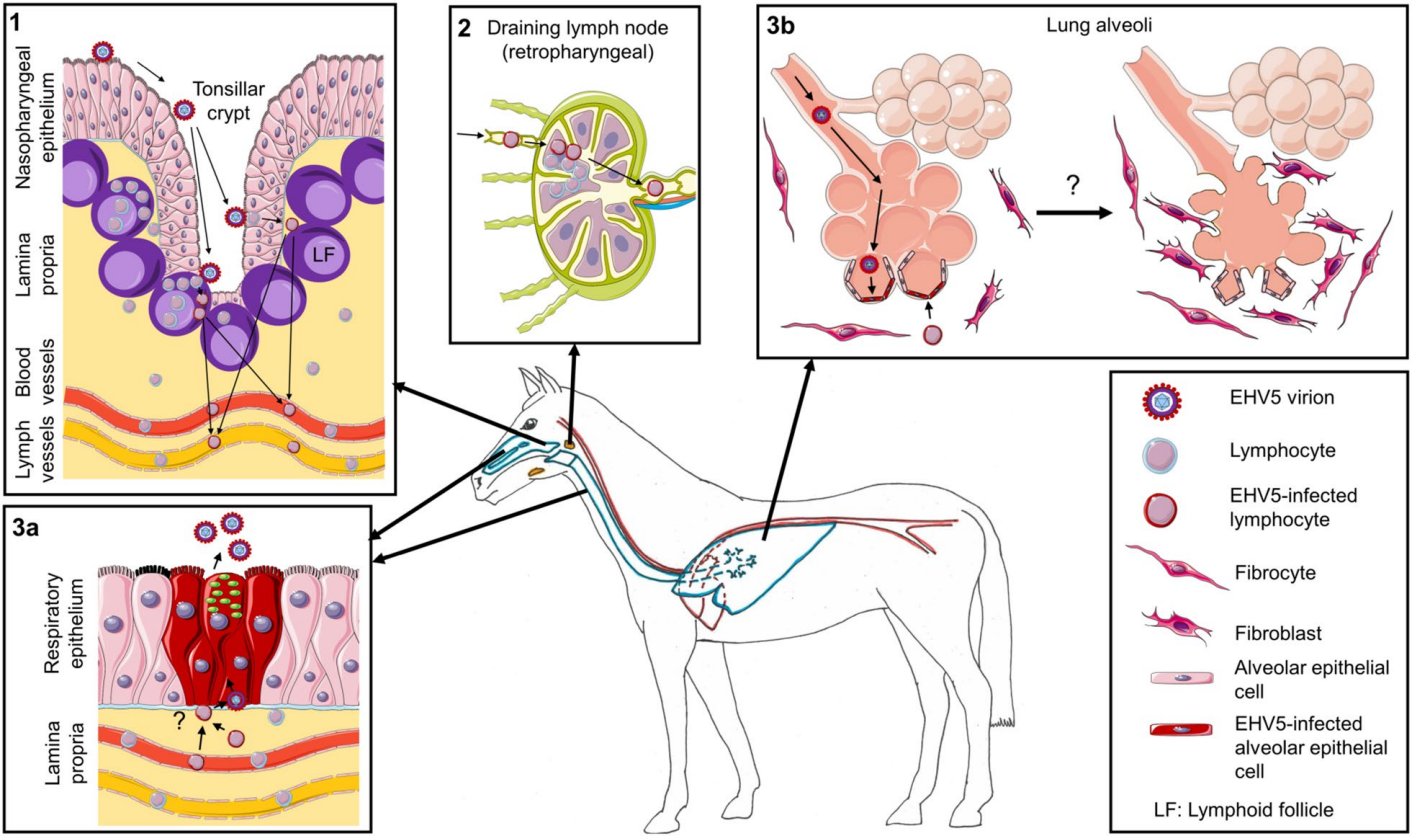
**Hypothetical model of EHV5 pathogenesis in the horse.** Drawings are based on SMART servier medical art templates. The horse’s respiratory tract is designated in blue, the circulatory system in red and the upper airway lymph nodes in orange. (1) EHV5 virions are propelled by the mucociliary escalator towards the tonsillar crypts, embedded in the nasopharynx. Here, EHV5 directly infects lymphocytes residing in lymphoid follicles (LF). Infected lymphocytes then transport the virus either directly to the bloodstream or via the lymph vessels and (2) the draining lymph nodes (especially the retropharyngeal lymph nodes) to the bloodstream. In the lymphoid follicles or draining lymph nodes, EHV5 spreads to neighbouring lymphocytes via cell–cell transfer. EHV5-infected lymphocytes might either succumb due to apoptosis or survive and function as a life-long reservoir for EHV5. Via the bloodstream or via lymphocyte-homing, EHV5-infected lymphocytes (re)route to different parts of the respiratory tract, e.g. the nasal cavities or the trachea (3a) or the lungs (3b). (3a) EHV5-infected lymphocytes might transfer infection to epithelial cells, which could amplify the infection and shed a high viral load in respiratory secretions. (3b) EHV5 infects alveolar cells and spreads to neighbouring cells via cell–cell transfer. Viral replication, together with host-specific predisposing factors might eventually trigger the onset of fibrosis and EMPF due to yet unknown reasons.

Based on this work, we suggest the following hypothetical model for EHV5 pathogenesis in the horse (Figure 6). Upon inhalation in a healthy horse, infectious EHV5 particles do not infect the ciliated respiratory epithelium, but are rather propelled by the mucociliary escalator towards the tonsillar crypts, embedded in the nasopharynx [62–64]. Lymphocytes reside in lymphoid follicles, just underneath the squamous epithelium of tonsillar crypts. As this epithelium contains gaps throughout the crypt surface, EHV5 possibly can directly access susceptible T and B lymphocytes. Following viral replication, virus particles are contained within these cells to protect them from the outer hostile environment. One part of these infected lymphocytes will eventually succumb due to apoptosis. The other part may be “saved” by EHV5 to function as a life-long latency reservoir. Via periodic reactivation, a latently infected horse will recurrently shed progeny virus to the outer world. Indeed, viral DNA is frequently recovered from PBMC and nasal secretions of healthy horses [9, 10, 23, 27]. How exactly the virus escapes from these lymphocytes to shed progeny virus in respiratory secretions is currently unknown. As for EBV, infected leukocytes might (re)route to the respiratory tract and produce virions lacking gp42 [58, 60]. These virus particles might then be transferred to epithelial cells, which could amplify the infection and shed a high viral load in respiratory secretions to infect new hosts. Finally, epithelial cell-derived EHV5 particles are optimally designed, i.e. contain gp42, to infect lymphocytes. Regular patrolling within the mucosa-associated lymphoid tissue (MALT) of latently-infected lymphocytes brings them to different sites of the respiratory mucosa, including the small bronchioli within the lungs. Here, free virus particles are able to infect alveolar cells and further spread to neighbouring cells using cell–cell transfer. Viral replication, together with host-specific predisposing factors (e.g. age and immunologic response) might eventually trigger the onset of fibrosis and EMPF [65]. Overall, our findings established the foundations for future research, which will eventually elucidate the mechanisms regulating EHV5 disease and triggering the development of EMPF.

## Additional files

**Additional file 1.** **Flow cytometric analysis of the equine T and B lymphocyte populations’ purity.** Equine T and B lymphocytes were diluted in PBS, containing 10% negative goat serum and antibodies (1:20) for 1 h at 4 °C. Equine T and B lymphocytes were incubated with a mouse monoclonal anti-CD3 antibody (clone UC_F6G) or a mouse monoclonal anti-pan B lymphocyte antibody (clone CVS36), respectively. The mouse monoclonal anti-PCV2 antibody (A27) was used as isotype (IgG1) control antibody. After a centrifugation step, cells were incubated with a goat anti-mouse IgG FITC^®^-conjugated antibody for 1 h at 4 °C. Finally, cells were analysed with a CytoFLEX flow cytometer (Beckman Coulter Life Sciences, Indianapolis, USA).

**Additional file 2.** **Validation of the polyclonal horse anti-EHV5 antibody (Sultan).** The biotinylated polyclonal horse anti-EHV5 antibody (Sultan) recognizes EHV5 antigens in EHV5-infected RK13 cells 48 hpi in both immunofluorescence (A) and immunocytological staining (B) (left panels). The biotinylated polyclonal horse anti-EHV1 antibody was included as control antibody (right panels). The scale bars represent 50 μm.

## Abbreviations

BoHV: Bovine herpesvirus
CO_2_: carbon dioxide
CPE: cytopathic effect
EBV: Epstein–Barr virus
EGTA: ethylene glycol tetra-acetic acid
EHV: equine herpesvirus
EMA: ethidium monoazide bromide
EMPF: equine multinodular pulmonary fibrosis
EREC: equine respiratory epithelial cells
FCS: fetal calf serum
FITC: fluorescein isothiocyanate
g/gp: glycoprotein
HHV: human herpesvirus
hpi: hours post-inoculation
Ig: immunoglobulin
IPMA: immunoperoxidase monolayer assay
MACS: magnetic-activated cell sorting
MALT: mucosal-associated lymphoid tissue
MHC: major histocompatibility complex
MOI: multiplicity of infection
MuHV: murine herpesvirus
NGS: negative goat serum
PBMC: peripheral blood mononuclear cells
PBS: phosphate-buffered saline
PFA: paraformaldehyde
pi: post-inoculation
RK13 cells: rabbit kidney epithelial cells
RT: room temperature
SD: standard deviation
TBS: tris-buffered saline
TCID_50_: tissue culture infectious dose with a 50% endpoint
TEER: trans-epithelial electrical resistance
VN: virus neutralizing
