# Supplementary figures and images for: Unravelling the first key steps in equine herpesvirus type 5 (EHV5) pathogenesis using ex vivo and in vitro equine models
Source: Vet Res. 2019 Feb 18;50:13. doi: 10.1186/s13567-019-0630-6 (PMC6380010; doi:10.1186/s13567-019-0630-6)

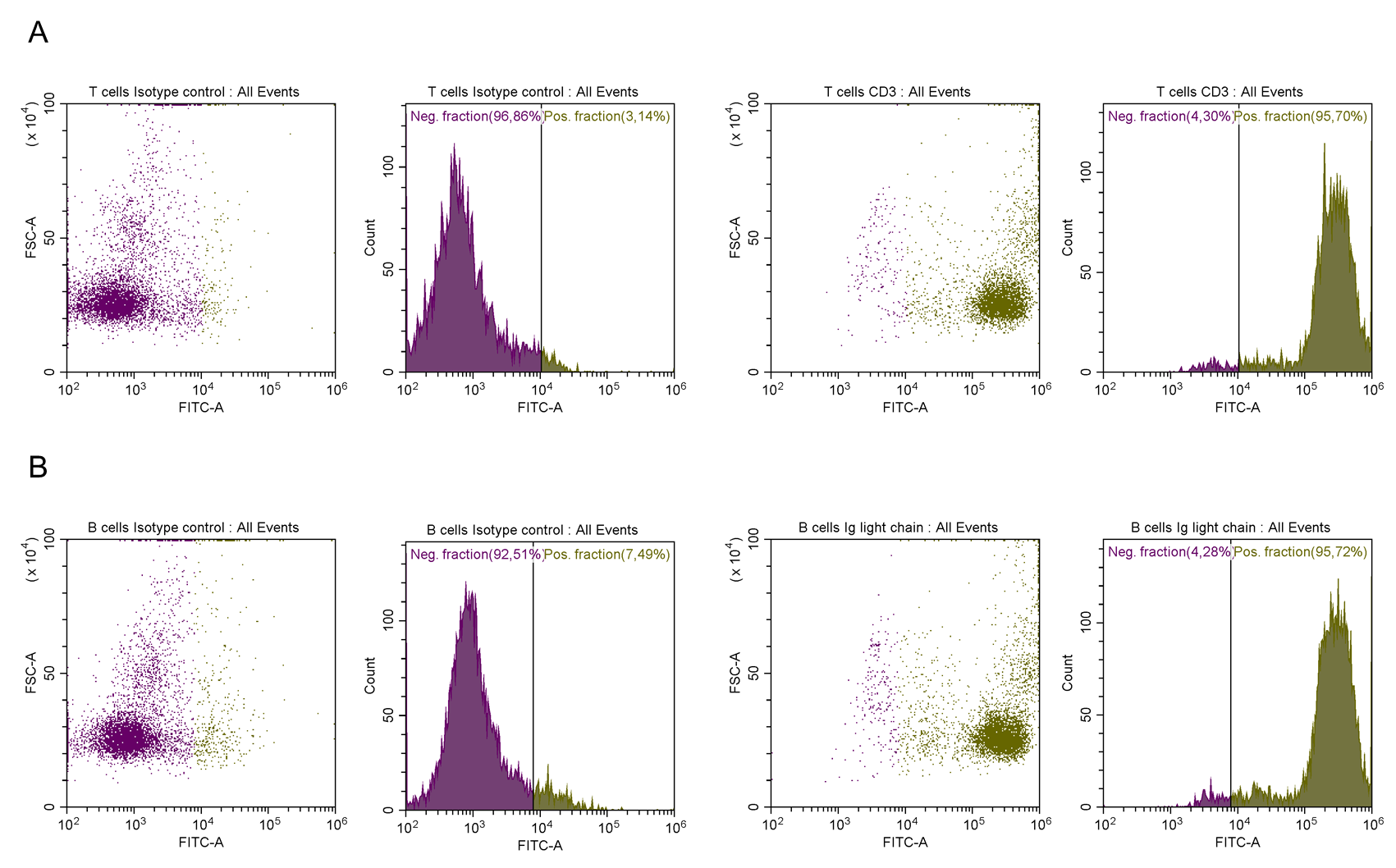

Supplement: Supplementary file 1 — Additional file 1. Flow cytometric analysis of the equine T and B lymphocyte populations’ purity. Equine T and B lymphocytes were diluted in PBS, containing 10% negative goat serum and antibodies (1:20) for 1 h at 4 °C. Equine T and B lymphocytes were incubated with a mouse monoclonal anti-CD3 antibody (clone UC_F6G) or a mouse monoclonal anti-pan B lymphocyte antibody (clone CVS36), respectively. The mouse monoclonal anti-PCV2 antibody (A27) was used as isotype (IgG1) control antibody. After a centrifugation step, cells were incubated with a goat anti-mouse IgG FITC®-conjugated antibody for 1 h at 4 °C. Finally, cells were analysed with a CytoFLEX flow cytometer (Beckman Coulter Life Sciences, Indianapolis, USA). [file 13567_2019_630_MOESM1_ESM.tif]

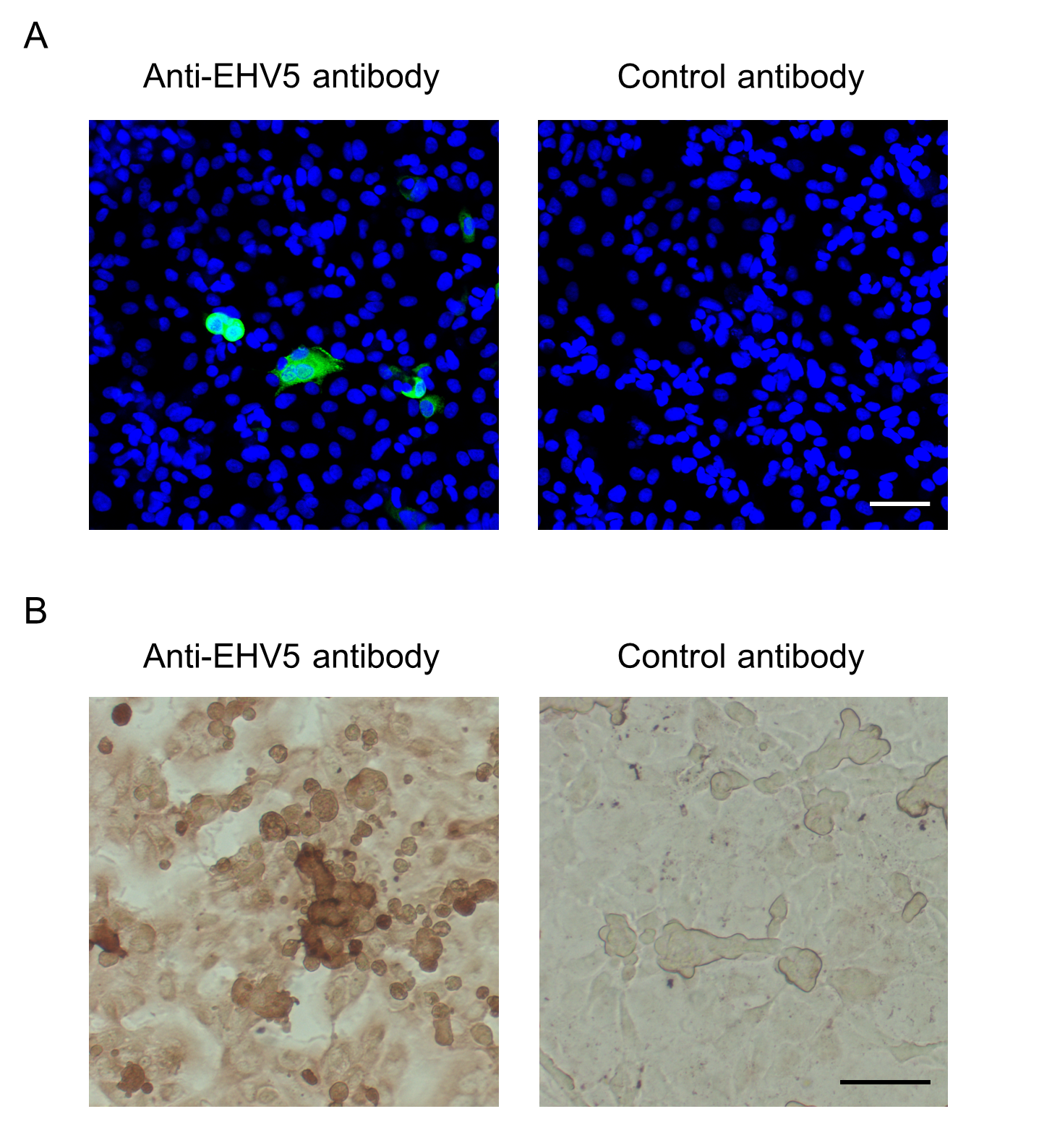

Supplement: Supplementary file 2 — Additional file 2. Validation of the polyclonal horse anti-EHV5 antibody (Sultan). The biotinylated polyclonal horse anti-EHV5 antibody (Sultan) recognizes EHV5 antigens in EHV5-infected RK13 cells 48 hpi in both immunofluorescence (A) and immunocytological staining (B) (left panels). The biotinylated polyclonal horse anti-EHV1 antibody was included as control antibody (right panels). The scale bars represent 50 μm. [file 13567_2019_630_MOESM2_ESM.tif]
